# Trans Fat Free by 2023—A Building Block of the COVID-19 Response

**DOI:** 10.3389/fnut.2021.645750

**Published:** 2021-03-24

**Authors:** Simone Bösch, Lucinda Westerman, Nina Renshaw, Igor Pravst

**Affiliations:** ^1^SICABO Consulting, London, United Kingdom; ^2^NCD Alliance, Geneva, Switzerland; ^3^Nutrition and Public Health Research Group, Nutrition Institute, Ljubljana, Slovenia

**Keywords:** COVID-19, trans fat elimination, trans fatty acids, NCD prevention, food regulation, cardiovascular diseases, non-communicable disease, artificial trans-fatty acids

## Abstract

COVID-19 has brought to center stage the most important health issue of our era, largely ignored by policymakers and the public to date: non-communicable diseases (NCDs), the cause of 71% of deaths per year worldwide. People living with NCDs, and particularly those living with cardiovascular disease (CVD), are at higher risk of severe symptoms and death from COVID-19. As a result, the urgent need for policy measures to protect cardiovascular health is more apparent than ever. One example of “low-hanging fruit” in the prevention of CVD is the elimination of industrially-produced trans fatty acids (iTFA). Their removal from the global food supply could prevent up to 17 million deaths by 2040 and would be the first time an NCD risk factor has been eliminated.

## Introduction

COVID-19 has illustrated the importance of public health and disease prevention measures, not only for infectious diseases, but also for NCD prevention and care. It is now recognized that COVID-19 and NCDs, often referred to as “underlying conditions,” are deeply linked. It is estimated that 1.7 billion people worldwide are at an increased risk of severe COVID-19 should they become infected, mostly as a result of living with one or more NCDs (1). These vulnerabilities include diet-related NCDs such as CVD and type 2 diabetes which are to a large extent preventable. The deadly interplay of NCDs, inequities and COVID-19 has illuminated the need to respond to the current crisis by breaking down silos and addressing interlinkages as a syndemic [formed from “syn-” = together and (epi)demic].

### The Syndemic of COVID-19 and NCDs

COVID-19 and NCDs interact to form a syndemic—parallel epidemics of health problems which interact synergistically, have intertwined risk factors and mutually enhance each other against a background of shared social and economic inequalities (2). The COVID-19 pandemic is occurring against the backdrop of a steadily rising NCD burden. NCDs are the leading cause of mortality worldwide with 41 million deaths annually, of which 32 million occur in low- and middle-income countries (LMICs) and 18 million are due to CVD (2, 3). NCDs and COVID-19 share factors which influence health-seeking behavior, health decision-making, access to healthcare and other services, and risk exposure: poverty, discrimination, cultural norms and gender (4).

NCDs and infectious diseases have often been addressed in silos, yet their interlinkages are well-known (5). Infectious diseases can be a risk factor for several NCDs, such as HIV and chlamydia for CVD (6), while NCDs increase the susceptibility to and disease severity of infectious diseases. NCDs were a predictor of disease severity for Middle East Respiratory Syndrome (MERS) and Severe Acute Respiratory Syndrome (SARS) (7, 8). Consequently, the 2018 High-Level Political Declaration on NCDs (9) called for the integration of responses to NCDs and infectious diseases.

COVID-19 has also worsened the obesogenic environment by limiting opportunities for physical activity and decreasing food quality for many, thus negatively impacting two of the main NCD risk factors (10, 11). Access to fresh food has become more limited for many. Lockdown measures and a decline in purchasing power increase reliance on cheap foods and foods with a long shelf life, both of which are often ultra-processed and unhealthy. In many countries, consumers have been targeted with new, unhealthy offerings by the food industry, with marketing messages tailored to exploit the pandemic context (12). Decreased dietary quality may persist even after COVID-19 is under control due to economic pressures in the pandemic's aftermath (10–14).

### COVID-19—A Historic Opportunity to Scale Up Health-Promoting Policy Measures

The interlinkage between infectious diseases, health emergencies and NCDs has brought about an unprecedented acknowledgment and visibility of the urgent need to address the ever-growing NCD burden. Unchecked, NCDs cause social and economic harm that far exceeds the damage caused by COVID-19. COVID-19 presents a historic imperative to prioritize and invest in public health by adopting health-promoting policy measures, including iTFA elimination. These measures must also address modifiable risk factors—including nutrition, hypertension and obesity—that drive both COVID-19 and the NCD burden.

Population groups of lower socio-economic status tend to consume higher amounts of iTFA and are therefore at higher risk of iTFA-attributable CVD. iTFA elimination can thus help reduce both CVD mortality and morbidity as well as health inequalities (15–18).

### Nutrition Policy to Address the Modifiable Risk Factors of NCDs

Currently, almost all countries are off-track to achieve the World Health Organization's target of reducing overall mortality from the four main NCDs—CVD, cancers, diabetes, and chronic respiratory diseases—by 25% by 2025 (19), and Sustainable Development Goal 3.4 to reduce premature mortality from NCDs by a third by 2030 (20, 21). Implementing strong nutrition policies will save lives, accelerate progress toward these global NCD targets, and build healthier, more equitable and resilient populations that are better prepared to deal with future health emergencies.

Nutrition policy interventions are one of the reasons high-income countries have managed to reduce CVD deaths by more than 25% since 2000 (22). Conversely, LMICs largely have yet to introduce comprehensive CVD prevention policies and bear up to 90% of the global CVD burden (23), underscoring the need to extend iTFA elimination strategies globally. This is particularly relevant in countries where Universal Health Coverage (UHC) does not yet exist, or is weak, and where primary prevention strategies such as nutrition policies can support the feasibility and sustainability of UHC.

Regulations such as mandatory iTFA limits link political will to health policy and demonstrate government commitment to addressing population health. Their adoption signals that a government is prepared to invest appropriately in public health, creates a level playing field for industry, and is a strong signal to society that a healthy diet and diet-related NCDs must be taken seriously.

## What are Trans Fats?

Trans fatty acids, or trans fats, are unsaturated fatty acids of either natural or artificial origin. Naturally occurring trans fats are produced by bacteria in the gut of ruminants; dairy and meat products derived from them contain small amounts of trans fats. iTFA are created in an industrial process that adds hydrogen to vegetable oil (hydrogenation) to produce partially hydrogenated oils (PHO), which are solid or semi-solid fats.

Globally, most iTFA is consumed through PHO which are common in baked goods, pre-packaged foods and some cooking oils. iTFA have no known health benefit and are a contributor to CVD worldwide, estimated to cause around 260,000 deaths and 6,162,986 disability-adjusted life years (DALYs) annually (24). Trans fat consumption increases the risk of death from any cause by 34% and from coronary heart disease (CHD) by 28% (25). For every 1% increase in daily energy obtained from trans fats, CHD mortality raises by 12% (18). iTFA intake has also been associated with an increased risk for other NCDs and related conditions such as ovarian cancer (26), infertility, endometriosis, Alzheimer's disease, diabetes and obesity (27, 28).

iTFA consumption induces low-grade systemic inflammation and is positively associated with endothelial dysfunction (a non-obstructive coronary artery disease without blockages of heart arteries, but with the large blood vessels of the hearts surface constricting instead of dilating) (29–33). Low-grade systematic inflammation, a higher concentration of pro-inflammatory cytokines and endothelial dysfunction are also induced by overweight and obesity which are metabolic risk factors for diet-related NCDs, and particularly for heart disease (34, 35). This is relevant in the context of COVID-19 which is a disease that triggers pro-inflammatory cytokines. Patients with severe COVID-19 frequently show cytokine storms, an excessive and uncontrolled release of pro-inflammatory cytokines; cytokine storms are an indicator for poor prognosis of COVID-19 (34, 36).

WHO recommends that total trans fat intake does not exceed 1% of total energy intake, which translates to >2.2 g/day for a 2,000-calorie diet (37).

iTFA can be replaced in foods with healthier fats and oils containing polyunsaturated (preferred) or monounsaturated fats without impacting their consistency and taste (38).

## Benefits of iTFA Elimination

Worldwide iTFA elimination could save 17 million lives by 2040 (39). Countries that have eliminated iTFA from their food supply have seen substantial health benefits:

- **Argentina:** iTFA elimination is associated with an estimated annual 1.3–6.3% reduction in CHD events (40).
- **Denmark**: In the 3 years following the implementation of an iTFA limit in 2004, CVD mortality decreased 3.2% in relation to comparable countries without iTFA regulation (41).
- **England and Wales**: iTFA elimination across the two countries is estimated to result in around 1,600 fewer deaths and 4,000 fewer hospital admissions per year (18).
- **New York**: Counties in the state of New York with iTFA restrictions saw 7.8% fewer hospital admissions for heart attacks between 2007 and 2013 than counties without iTFA restrictions (42).

The prevention of death and disease attributable to iTFA consumption lessens the burden on health systems, which is particularly important for health facilities overwhelmed by the COVID-19 response and where treatment services for CVD and other NCDs have been disrupted.

The economic value of investing in global iTFA elimination has not been calculated but local estimates demonstrate the intervention's cost-effectiveness.

- **Argentina**: iTFA elimination would save US$17-87 million annually in costs associated with the management of CHD complications and follow-up. These cost savings include implementation costs of the policy incurred by the Ministry of Health, but do not include other economic costs (e.g., lost productivity due to CVD) (40).
- **Australia**: iTFA elimination would save AU$80 million (US$60 million) in healthcare costs related to ischemic heart disease during the first 10 years and AU$538 (US$407 million) over the population lifetime. Policy costs would near AU$22 million (US$17 million) during the first 10 years and AU$56 million (US$42 million) over the population lifetime, mostly consisting of monitoring costs to government (17).
- **European Union**: Prior to adopting a mandatory 2% iTFA limit, the European Union estimated that phasing out iTFA would result in direct and indirect cost savings of €58–304 billion (US$68–358 billion) over 85 years (16).
- **United Kingdom**: One study found that iTFA regulation in England would result in cost savings of around £297 million (US$379 million), consisting of £42 million (US$54 million) in direct healthcare costs, £196 million (US$250 million) in informal care costs, and £59 million (US$75 million) in averted productivity loss over 5 years. Considering implementation costs to government and industry, net cost savings would range from £64–264 million (US$82–337 million) (15). Another study calculated that mandatory iTFA elimination in England and Wales over a 10-year period would bring cost savings of £755 million to £1.54 billion (US$965 million to US$1.97 billion), comprising £95–201 million (US$121–257 million) in direct healthcare costs, £368–727 million (US$470–929 million) in informal care costs, and £292–613 million (US$373–783 million) in averted productivity loss (18).
- **United States**: The removal of PHO over a 20-year time interval is estimated to result in net benefits of US$130 billion. The analysis included lives saved and non-fatal illnesses prevented as benefits, and as costs product reformulation and relabelling, increased costs of substitute ingredients, costs to consumers from changing recipes, reduced product acceptances, shorter product shelf life, and restaurants and bakeries learning how to operate without PHO (43).

WHO deems iTFA elimination a cost-effective and feasible intervention (a so-called “best buy” policy measure), recommended for implementation by all countries to prevent NCDs (44).

## WHO's REPLACE Initiative

**Figure 1:**
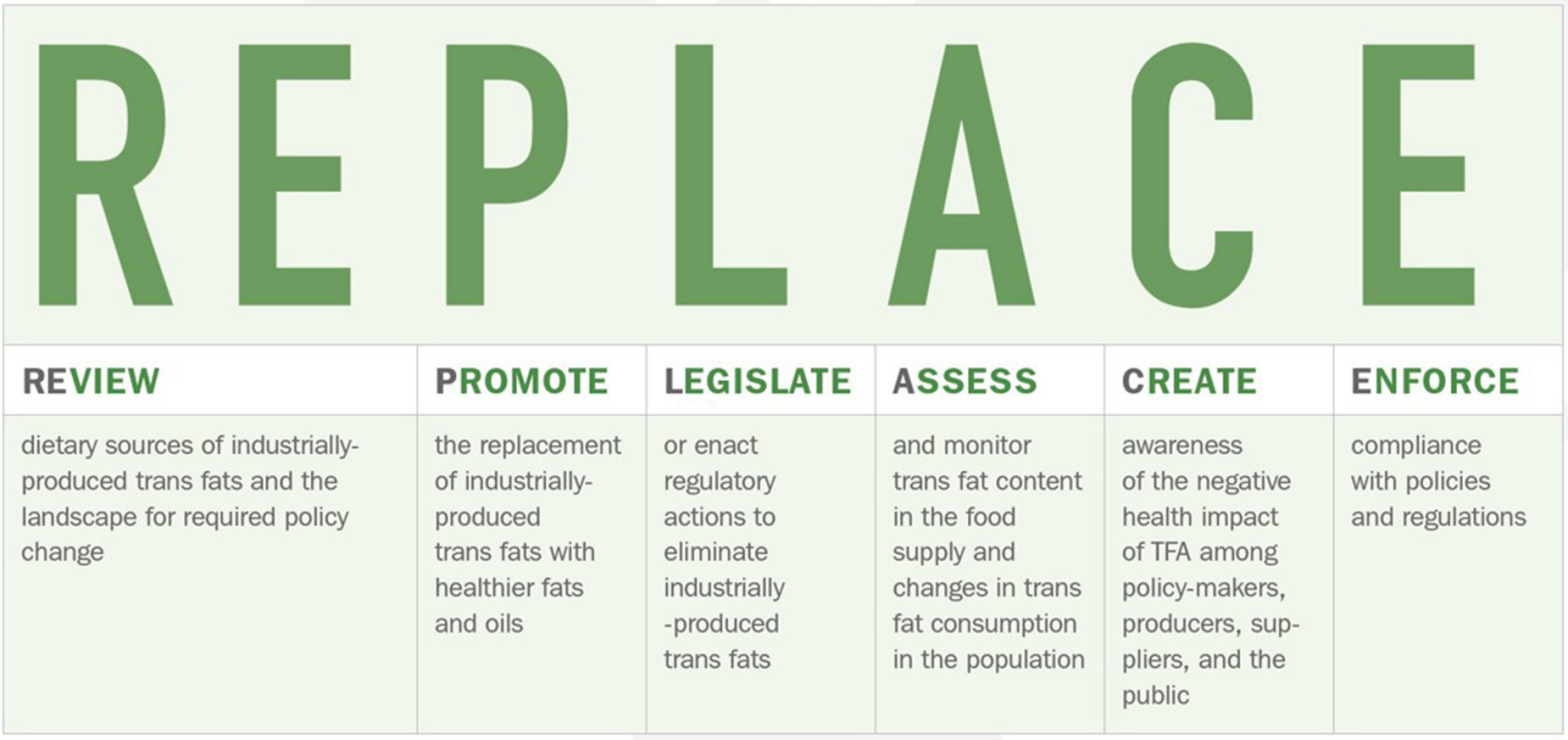
The six areas of WHO's REPLACE action package to eliminate iTFA (46).

To support national governments to reach the goal of global iTFA elimination by 2023, WHO launched the REPLACE initiative in May 2018. The REPLACE action package (45) provides governments with evidence-based tools across six strategic areas to eliminate iTFA from their national food supply (see Figure 1). REPLACE is the first global initiative to eliminate an NCD risk factor. In September 2020, WHO announced a certification scheme which will recognize countries that achieve iTFA elimination, similar to the WHO certification scheme for polio eradication (47). Countries must show that they have implemented a best-practice iTFA policy and that effective monitoring and enforcement is in place to qualify for certification (48). This initiative is the first time that WHO has introduced certification to recognize government's achievements in addressing a modifiable NCD risk factor.

## 3.2 Billion People will be Protected by iTFA Policies by 2021 but Over 100 Countries Have Yet to Act

Substantial progress has been made in the last 10 years to remove iTFA from the global food supply. To date, 32 countries have enacted laws and regulations that currently protect 2.4 billion people from this harmful substance. Another 26 countries have passed laws and regulations that will come into effect in the next 2 years, covering a further 815 million people. Encouragingly, an increasing number of countries are introducing best practice policies, which include setting a compulsory limit of 2 g of iTFA per 100 g of total fat/oil in all foods and/or banning PHO (the main source of iTFA). While in 2010 only two countries had a best practice policy in effect, this number has risen to 14 by 2020 and will reach 40 by 2022 (49). These developments show that adopting legal instruments to limit iTFA or ban PHO is politically, economically, and technically feasible (50).

Regional approaches to iTFA elimination have also progressed. Member States of the Pan American Health Organization (PAHO, WHO's Americas region) unanimously approved a Regional Plan of Action to Eliminate Industrially Produced Trans-Fatty Acids 2020–2025 (51), the first of its kind globally. Regional regulations include the European Union's 2% iTFA limit for all foods adopted in 2019 (52), a Gulf Cooperation Council standard limiting iTFA to 2% for fats and oils and 5% for other foods in 2015 (53), and the Eurasian Economic Union's 2% iTFA limit for oils and fats adopted in 2015 (54). Such regulatory approaches have the added benefit of spill-over effects, forcing countries surrounding these regions to consider iTFA elimination policies to allow for continued trade in foods.

However, over 100 countries have yet to act, and of the 15 countries with the highest proportion of CHD deaths due to trans fat intake, only four (Canada, Latvia, Slovenia, and USA) have introduced regulations to remove iTFA from their food supply. Ten countries (Azerbaijan, Bangladesh, Bhutan, Ecuador, Egypt, Iran, Mexico, Nepal, Pakistan, and Republic of Korea) have yet to do so, while India is on track for a best practice policy (49).

Countries with comparatively low iTFA intake and associated mortality also benefit from adopting iTFA regulations. Introducing regulation is a preventive measure to avoid increasing intakes of iTFA and associated health risks in the future, and to guard against food manufacturers increasing sales of iTFA-containing foods (“dumping”) in unregulated markets. Additionally, average iTFA intake levels at national level may conceal high iTFA exposure levels in pockets of the population—regulation ensures that health disparities due to iTFA intake are minimized. And implementation of iTFA regulation is easier and cheaper when national levels of iTFA are low, also presenting an opportunity to strengthen regulatory capacity and systems in food safety (55).

Disparities in protection from iTFA also persist. Most laws and regulations have been adopted in high-income or upper-middle-income countries in Europe and the Americas. No low- or lower-middle-income country has implemented a best practice policy to date, resulting in geographic and socio-economic inequalities (49). This is particularly worrying given that CVD associated mortality is higher in LMICs than high-income countries (56).

## Mandatory Regulation is Preferable to Voluntary Commitments to Phase Out iTFA

In 2019, member organizations of the International Food & Beverage Alliance (IFBA) committed to limit iTFA to 2 g per 100 g fat/oil in their food products worldwide by 2023 and to reformulation without increasing the content of saturated fat (57). It will be important that adherence to and impact of these commitments is independently and transparently monitored and evaluated.

In the 2008 Trans Fat Free Americas Declaration (58), backed by PAHO, representatives of Latin America's major food companies (including some IFBA members), cooking oil companies and industry associations, together with delegates of national public health authorities, committed to a 2% iTFA limit in oils and margarines and a 5% limit in other foods.

These voluntary efforts, however, only cover a small percentage of packaged foods worldwide (49), and the food industry and suppliers of oils and fats have generally been slow to voluntarily phase out iTFA. Many large food producers have replaced iTFA with healthier fats in products sold to high-income countries—many of which have regulated iTFA—while resisting the replacement of iTFA in LMICs (49, 59).

Food industry and oil and fat suppliers may be reluctant to phase out iTFA for fear of competitors moving into the market if regulation is absent to create a level playing field. Additionally, compliance with voluntary commitments cannot be enforced by governments. Research shows that voluntary approaches are less effective than mandatory regulation in reducing iTFA content in foods (28, 60). Therefore, compulsory regulation combined with strong enforcement mechanisms is recommended over voluntary schemes.

## iTFA Elimination Should be Embedded in a Comprehensive Policy Approach

Diet is one of the key modifiable risk factors to address underlying conditions of severe COVID-19. Therefore, including iTFA elimination in a comprehensive policy approach to improve the food environment will address both NCDs and the ongoing pandemic. It will also improve preparedness for and resilience to future pandemics, as a healthier population with a lower prevalence of NCDs is less susceptible to infections and better equipped to fight them.

In addition to iTFA regulation, a comprehensive policy package to prevent diet-related NCDs should comprise mandatory food labeling (ingredient lists, nutrient panels declaring trans fats, interpretative front-of-pack labeling based on nutrient profiles, rules on nutrient and health claims), restrictions on food marketing aimed at children and adolescents, mandatory standards for healthy school food, limits on salt/sodium content (61), and nutrition standards for public procurement. These policy measures can be accompanied by public awareness campaigns to educate consumers on healthy nutrition.

At the healthcare level, policy actions should include preventative measures such as blood pressure checks and hypertension control (62), weight-management support and nutrition counseling.

Additionally, taxing unhealthy foods and beverages alongside alcohol and tobacco—and removing any market-distorting subsidies—would reduce their intake and, in some cases, incentivize reformulation while mobilizing domestic revenue, which could be invested in health system strengthening and Universal Health Coverage. Such investments would not only contribute to future health, but also pandemic preparedness and health systems' resilience. Notably, if used progressively, such revenue would benefit poorer households and help tackle poverty and inequality. For example, raising the price of sugar-sweetened beverages, alcohol, and tobacco by 50% could raise around US$24.7 billion in 54 LMICs by 2030 (63).

## Conclusion

Including iTFA elimination alongside a comprehensive policy approach including food policy, healthcare and taxation strategies—many of them WHO “best buys”—in recovery packages will strengthen global health systems, as compared to pre-pandemic levels. Using these population-wide primary prevention strategies in the COVID-19 response will serve as a stepping stone to tackle the world's biggest killer, cardiovascular disease; support economic recovery from the pandemic; and increase health security by making future generations more resilient to infectious diseases.

## Data Availability Statement

The original contributions presented in the study are included in the article/supplementary material, further inquiries can be directed to the corresponding author/s.

## Author Contributions

This perspectives paper was conceived and written by SB. NR, LW, and IP reviewed the manuscript. All authors read and approved the final manuscript.

## Conflict of Interest

SB consults for both the NCD Alliance and Resolve to Save Lives. NR and LW are employees of NCD Alliance. IP led and participated in various research projects in the area of nutrition, public health, and food technology, including a project “Trans fats in foods,” funded by the Slovenian Research Agency, Ministry of Health of the Republic of Slovenia, and food businesses.
